# Reconstruction of the Sternoclavicular Joint With an Autologous Fibular Head: A Case Report

**DOI:** 10.1002/ccr3.70919

**Published:** 2025-10-27

**Authors:** Yuan Gao, Feng Pan, Jun Zhang, Songjie Ji, Guanning Shang

**Affiliations:** ^1^ Department of Joint Surgery Guizhou Hospital of Beijing Jishuitan Hospital Guiyang Guizhou Province People's Republic of China; ^2^ Department of Orthopaedic Surgery Beijing Jishuitan Hospital, Capital Medical University Beijing People's Republic of China; ^3^ Department of Bone and Soft Tissue Tumor Surgery Shengjing Hospital, China Medical University Shenyang Liaoning Province People's Republic of China

**Keywords:** case report, fibular head autograft, functional evaluation, sternoclavicular joint reconstruction, sternocleidomastoid muscle reconstruction

## Abstract

Medial clavicle defects resulting from medial clavicle resection may lead to related complications and pain in patients. Additionally, the clavicle acts as a shield to protect the neurovascular structures located below the medial third; therefore, it is recommended to reconstruct the medial clavicle after resection. We report a unique case of autologous fibular head transplantation for sternoclavicular joint reconstruction, with the assistance of three‐dimensional (3D) reconstruction and mixed reality (MR) technology. A 21‐year‐old female patient presented to the hospital with a painless mass in the right medial clavicle. The mass gradually grew larger and became painful during the following 2 months. There was no previous history of tuberculosis, fever, weight loss, or night sweats. Computed tomography angiography showed bone destruction and a soft tissue mass, with the lesion resembling a malignant bone tumor. Using preoperative 3D reconstruction and MR technology, the articular surfaces of the fibular head and clavicle were found to have a certain degree of similarity. Therefore, sternoclavicular joint reconstruction using the fibular head graft was performed. At the 1‐year follow‐up, the clavicular pain was completely relieved, the Constant–Murley score was improved, and the cosmetic effect was satisfactory. Current methods of clavicular reconstruction mainly include biotic and abiotic reconstruction. Among these, biotic autologous bone is a good material for the repair and reconstruction of bone defects. Three‐dimensional reconstruction and MR can provide spatial visualization. In this case, this combination allowed complete resection and subsequent effective reconstruction.

**Summary**

- This study reports a unique case of autologous fibular head transplantation for sternoclavicular joint reconstruction in a patient with a medial clavicular tumor, with the assistance of three‐dimensional (3D) reconstruction and mixed reality (MR) technology.
- We found that, in this case, the combination of 3D and MR promoted effective reconstruction of the sternoclavicular joint, with satisfactory clinical results after surgery.
- We believe that our study makes a significant contribution to the literature because the combination of 3D and MR allowed us to successfully remove the medial clavicular lesions without damaging the subclavian nerves and vessels.
- Moreover, it also demonstrates the feasibility of using an autologous fibular head to reconstruct the sternoclavicular joint and restore the acromioclavicular joint range of motion.

## 1 Introduction

Recurrent clavicular tumors and tumor‐like lesions are rare, accounting for about 1% of all bone tumors [1]. Among these, chondrosarcoma is a common primary clavicular malignant tumor, which usually reaches a safe surgical boundary through segmental resection. However, bone defects caused by clavicular tumor resection may trigger related complications, especially the medial clavicular defect, which instigates pain in patients. Therefore, Nistor et al. [2] advocated reconstruction after medial clavicular resection to restore the normal shape and function of the clavicle and prevent shoulder joint dysfunction. Furthermore, aesthetics is also a potential concern after total or partial claviculectomy, particularly in female patients [3]. Three‐dimensional (3D) reconstruction and mixed reality (MR) technology play key roles in the surgical treatment of bone tumors. Three‐dimensional reconstruction integrates two‐dimensional images into 3D and allows rotation according to clinical needs; therefore, the position and shape of lesions can be displayed intuitively and clearly. Furthermore, MR technology enables accurate matching between the 3D virtual model and the real world, which can improve the accuracy of orthopedic surgery and make the treatment plan more accurate and efficient. In this study, we describe the reconstruction of the sternoclavicular joint using the ipsilateral fibular head. We also detail how to use 3D reconstruction and MR technology to verify the feasibility of the reconstruction scheme. This report is based on the Surgical CAse REport (SCARE) checklist [4].

## 2 Case Presentation

### 2.1 Medical History

A 21‐year‐old female patient presented with a painful mass in the region near the right sternoclavicular joint. The mass had been increasing in size over the previous month and caused pain upon movement (mainly flexion and abduction) of the shoulder. The patient had no abnormal symptoms and denied fever, chills, night sweats, weight loss, and fatigue.

### 2.2 Physical Examination

Physical examination showed that the right clavicular skin surface temperature and color were normal. The 4.5 cm mass was oval in shape with a solid texture and was localized at the medial third of the clavicle and was extremely tender on palpation. The overlying skin was nonadherent and freely mobile. Active shoulder range of motion (ROM) was 130° on elevation, while internal and external rotation were preserved. The Constant–Murley shoulder outcome score, which evaluates pain, daily living activities, strength, and shoulder ROM, was 70/100.

### 2.3 Radiographic Assessment

**FIGURE 1:**
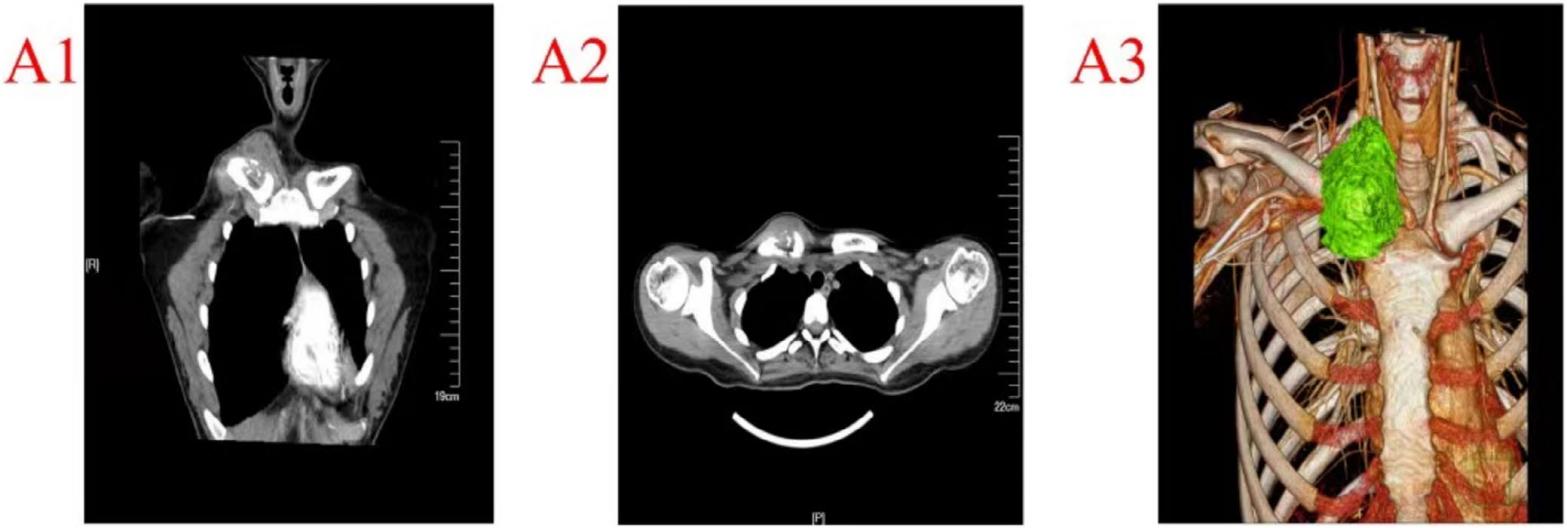
(A1–A3) Preoperative computed tomography angiography shows obvious tumor destruction of the medial end of the right clavicle and adjacent soft tissue erosion.

Computed tomography angiography (CTA) showed bone destruction and a soft tissue mass in the sternal segment of the right medial clavicle, sized approximately 46 mm × 32 mm × 38 mm, which was significantly clearer after enhancement. The boundary between the mass and the right subclavian artery was clear, and the sternoclavicular joint appeared to be spared (Figure 1). No lung metastasis was demonstrated by thin‐slice chest computed tomography (CT).

### 2.4 Preoperative Planning

**FIGURE 2:**
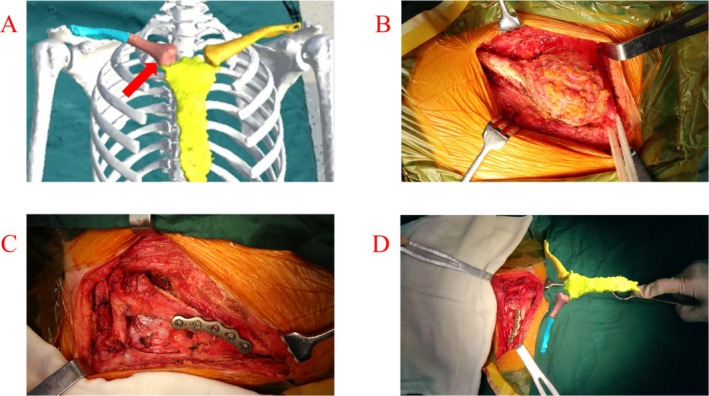
(A) The three‐dimensional virtual structures of the ipsilateral fibular head for reconstructing sternoclavicular joints are displayed. (B) An intraoperative photo showing that the tumor was fully dissociated. (C) Intraoperative photo: The fibular head and part of the biceps femoris tendon were sutured to the sternum with high‐strength No. 2 sutures. Additional stability was achieved by passing a high‐strength suture through the fibula into the upper surface of the adjacent first costal cartilage. The lateral collateral ligament attached to the fibular head was reconstructed, using absorbable sutures, to the remaining sternocleidomastoid muscle. (D) Comparison of virtual images provided by the intraoperative mixed reality technique with actual intraoperative reconstruction.

According to the clinical manifestations and CTA imaging findings, the involved bone was destroyed and accompanied by soft tissue masses; therefore, the focus tended toward primary malignant tumors. Total resection of the tumor, including the medial clavicle and portions of the sternocleidomastoid muscles, was performed before reconstructing the sternoclavicular joint and sternocleidomastoid muscle. Preoperatively, we discussed whether the ipsilateral fibular head with the lateral collateral ligament could be used for the reconstruction of the sternoclavicular joint and sternocleidomastoid muscle. Using 3D reconstruction and MR technology, we found that the clavicular joint surface could be substituted for the ipsilateral fibular head joint surface, with a certain degree of similarity between these (Figure 2A), and the lateral collateral ligament attached to the fibular head could be used to reconstruct the sternocleidomastoid muscle.

### 2.5 Surgery

#### 2.5.1 Stage 1 of the Surgery: The Ipsilateral Proximal Fibula Was Harvested With a Small Portion of Attached Ligament

The ipsilateral fibula with the required length was cut according to the preoperative plan. A longitudinal skin incision was made at the lateral side of the lower leg on the ipsilateral limb to expose the proximal end of the complete fibula. After confirming and carefully protecting the common peroneal nerve, the fibula was cut off approximately 6 cm from the fibular head, and the proximal end of the fibula was completely removed. While the proximal tibiofibular joint was free, the graft retained part of the lateral ligaments and the biceps femoris tendon attached to the fibular head. Subsequently, the remaining lateral collateral ligament and biceps femoris tendon were sutured and reconnected to the tibia. Hemostasis was achieved before closing the wound.

#### 2.5.2 Stage 2 of the Surgery: Surgical Treatment of the Clavicular Tumor

A 17‐cm incision was made on the surface of the clavicular tumor, the skin and subcutaneous tissue were cut, the deep fascia was separated layer by layer, and the tumor was fully exposed (Figure 2B). The surrounding blood vessels, tissues, and tumor infiltration in the medial clavicle were explored. We found that the medial clavicle and part of the sternocleidomastoid muscle were involved, requiring extensive resection.

#### 2.5.3 Stage 3 of the Surgery: Reconstruction of the Sternoclavicular Joint and Sternocleidomastoid Muscle

The sternum and clavicle were exposed, and the fibular head was trimmed for more similarity to the articular surface of the proximal clavicle. Subsequently, under the guidance of MR technology, the joint was reduced and its ends were secured with non‐absorbable high‐strength sutures. The fibular head and part of the biceps femoris tendon were also sutured to the sternum with high‐strength sutures. Subsequently, additional stability of the reconstructed joint was achieved by passing a high‐strength suture through the fibula into the upper surface of the adjacent first costal cartilage. Thereafter, the reconstruction plate was connected to the distal segment, and four cortical screws were used to fix the clavicle and the fibula. Dynamic stability of the sternoclavicular joint was then tested under direct visualization and palpation. The lateral collateral ligament attached to the fibular head was meticulously reconstructed with absorbable sutures and attached to the remaining sternocleidomastoid muscle (Figure 2C,D). Lastly, the subcutaneous layer and skin were closed in a standard fashion.

### 2.6 Postoperative Management

**FIGURE 3:**
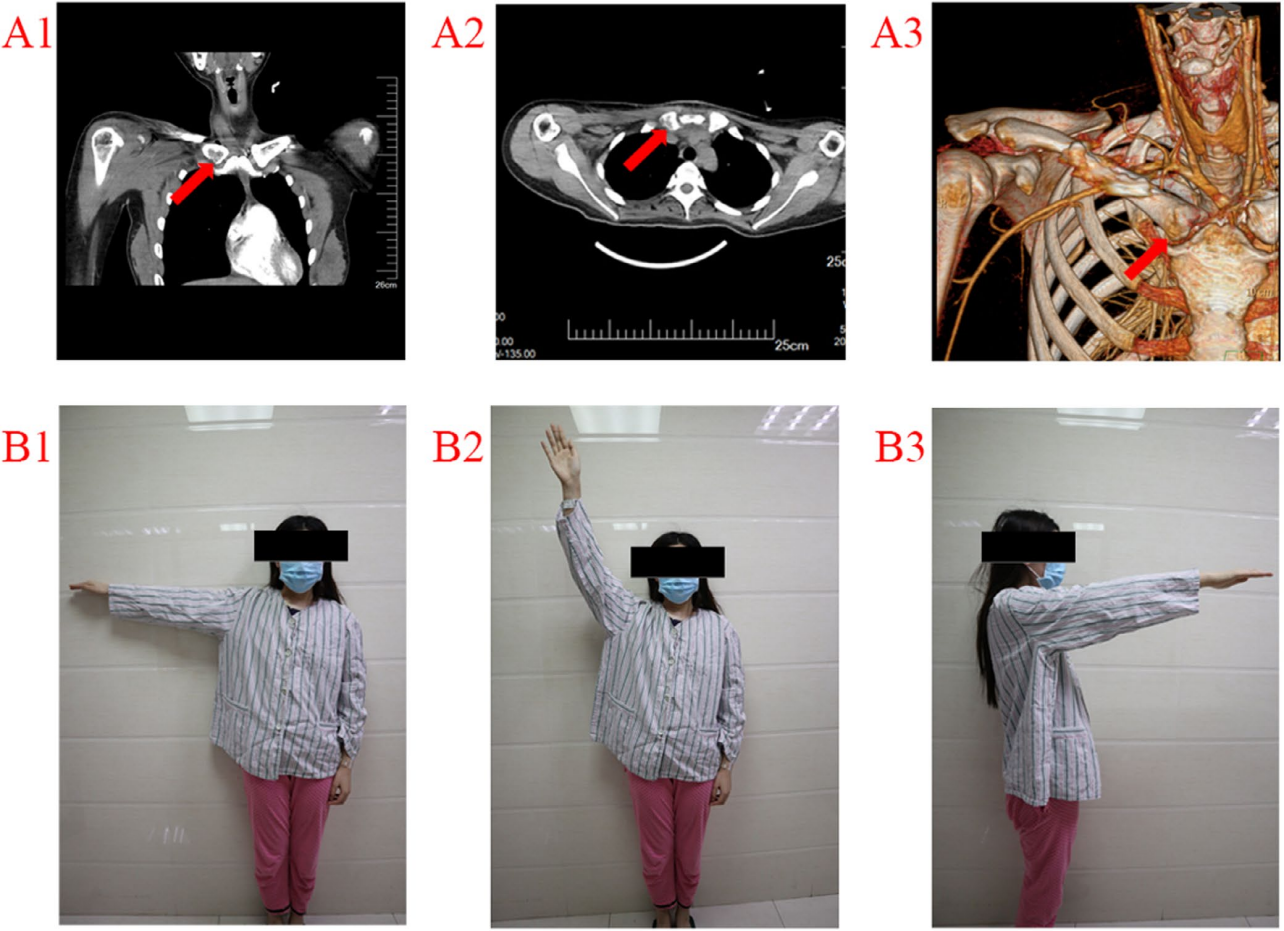
(A1–A3) Postoperative computed tomography scans show the reconstructed sternoclavicular joint. (B1–B3) A satisfactory range of motion of the shoulder after reconstruction of the sternoclavicular joint.

**FIGURE 4:**
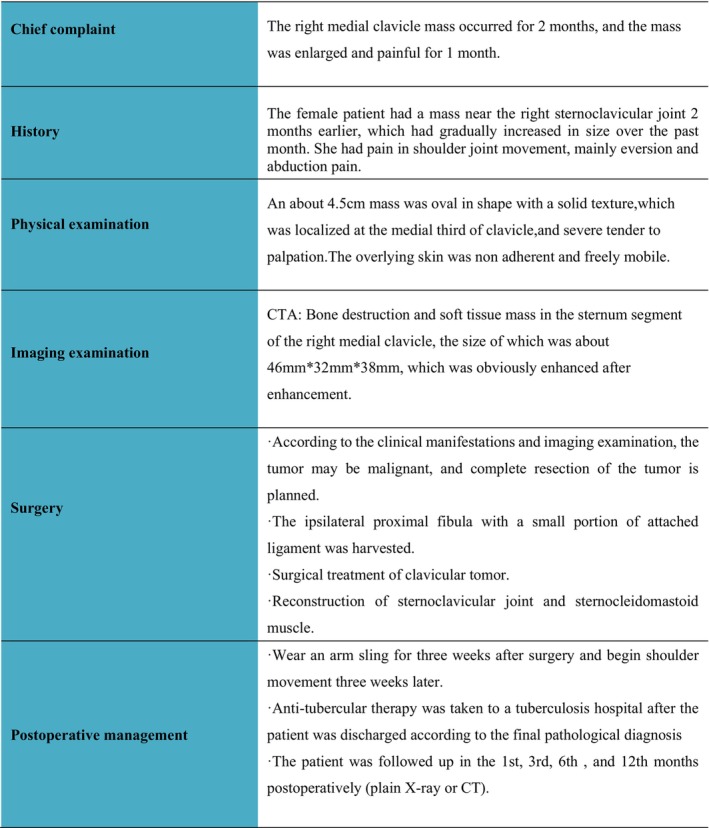
The therapeutic course followed by the patient. The current case report is presented based on this sequence of event.

No radiotherapy or chemotherapy was administered postoperatively, and wound healing was uneventful. Postoperative CT was obtained (Figure 3A1–A3), and the patient needed to wear an arm sling for 3 weeks postoperatively. The pathological diagnosis was chondrosarcoma. The patient was followed up in the 1st, 3rd, 6th, and 12th months postoperatively, using either plain X‐ray or CT scanning. At these follow‐ups, the range of shoulder movement, the degree of pain, tumor recurrence, and metastasis were evaluated. At the last follow‐up, 1 year postoperatively, the ROM of the right shoulder was normal, with no pain or signs of recurrence or metastasis (Figure 3B1–B3). The patient did not have any major immediate postoperative complications in the donor or recipient areas. The treatment course is summarized in Figure 4.

## 3 Discussion

Medial clavicular tumors, especially malignant tumors, are rare and can be controlled by segmental resection. However, this results in the loss of the sternoclavicular joint. It is well known that shoulder joint activities are based on the sternoclavicular joint as the fulcrum, with the clavicle as the lever; therefore, the lack of a medial clavicle may lead to shoulder instability [5]. In a study conducted by Green et al. [6], they noted that although medial clavicle resection is acceptable, biomechanical and clinical evidence suggests that the stability of the shoulder joint will be affected, which further leads to shoulder weakness, pain, lateral recumbent pain, abduction, and lift limitation of the affected limb, neurovascular bundle damage, shoulder drop, and other complications. Therefore, we believe that reconstruction after medial clavicle resection can restore the normal shape and function of the clavicle to the greatest extent possible, preventing shoulder joint dysfunction. Presently, the methods of clavicle reconstruction mainly include biotic and abiotic reconstruction. The biological reconstruction is usually performed with autologous ribs, the fibula, and large allogeneic bones [7, 8, 9, 10, 11], while the non‐biological reconstruction materials mainly use bone cement. The reconstruction of clavicle defects with bone cement is simple and rapid, and can effectively stabilize the acromioclavicular joint, but the long‐term effect is controversial [5]. Ai et al. [12] reported a case of nonunion in a 52‐year‐old woman after conservative treatment of a left clavicle fracture. Reconstruction was done through the medial femoral condyle. At the 4 months follow‐up, the patient was pain‐free and had recovered her mobility. However, this method is not suitable for long bone defects. Louie et al. [13] reported a case of a 52‐year‐old man with clavicular dermatofibroma who underwent surgical resection for clavicular reconstruction using a free vascularized fibula flap. The patient had a functional Constant–Murley score of 77 and a perfectly symmetrical ROM without pain. Rebecca et al. [14] described acute reconstruction of a 6.5‐cm bone gap after a gunshot wound to the chest in a 29‐year‐old man using a 6.5‐cm vascularized free fibula flap and periosteum extension to reconstruct a bone defect. ROM was complete and painless 8 months after surgery, suggesting that this technique may be a viable option after acute trauma. Devaraj et al. [15] reported on an 11‐year‐old woman with a pathological clavicle fracture due to a tumor. The clavicle defect was filled intraoperatively with a composite flap and a vascular rib graft. Three months after the operation, the patient had no paresthesia nor limited movement in the intervened area. Momberger et al. [9] previously reported the use of a bone allograft to reconstruct a bone defect after clavicle trauma; however, the graft was finally removed because of complications. The presence of infection and nonunion are risks in the use of allografts. Therefore, in our case, autologous bone reconstruction was the best reconstruction method owing to the comprehensive analysis of the patient's age, sex, and the nature of the lesions.

Only a few studies have analyzed the approaches to clavicular tumor resection and reconstruction, and most of these studies are case reports or include a small number of cases. To our knowledge, no previous studies have compared the outcomes of autologous bone reconstruction for clavicle defects and, as such, there is no certainty regarding which reconstruction method is better. In our study, we preferred fibular reconstruction because the cartilage surface of the fibular bone can reduce friction and the risk of arthritis. We reconstructed the sternoclavicular joint with an autogenous fibular head and plated internal fixation with the assistance of 3D reconstruction and MR technology to achieve ideal biomechanical stability. In order to ensure a safe surgical resection boundary, we cut the clavicular end of the sternocleidomastoid muscle from the bone attachment and reconstructed it with the fibular collateral ligament attached to the fibular head. This reconstruction aimed to avoid various adverse outcomes after surgery, including pain, limited mobility, and facial asymmetry [16]. Moreover, the sternoclavicular and costoclavicular ligaments were reconstructed with high‐strength sutures and an autologous tendon to maintain the stability of the sternoclavicular joint [17].

The spatial structure of clavicular tumors and their associated surrounding soft tissue structures can be visualized by 3D reconstruction [18]. Additionally, MR technology is used to transform 3D models into spatial models of virtual images, enabling easy identification and visualization of the relevant anatomical structures. Moreover, we can also simulate surgery and implant placement with MR technology [19]. The results showed that the fibular capitulum joint surface was similar to the original clavicular joint surface, and the feasibility of reconstructing the sternoclavicular joint surface was verified through simulated surgery. Therefore, 3D reconstruction and MR technology can facilitate reconstruction planning according to the size and shape of the bone defect, as well as help practice removing the tumor and placing the implant in advance [20]. During the operation, the reconstructed holographic visualization 3D modeling will be projected to the corresponding anatomical position of the human body, and the operator will be guided to place the graft and steel plate in the best position during the reconstruction process. The application of its “perspective” function in the operation area during the nail placement process can improve the accuracy and safety of screw placement, reduce the number of intraoperative X‐ray fluoroscopies, and shorten the operation time [21]. Moreover, visualization can provide patients and their families with an accurate and vivid understanding of the local anatomical structures, treatment methods, and surgical procedures; therefore, they can understand the treatment plan and potential risks of surgery more clearly and intuitively [22]. With the help of 3D reconstruction and MR technology, we successfully removed the medial clavicular lesions without damaging the subclavian nerves and vessels. Furthermore, we also demonstrated the feasibility of using an autologous fibular head to reconstruct the sternoclavicular joint and restore the ROM of the acromioclavicular joint. At the 1‐year follow‐up, the X‐ray film showed that the sternoclavicular joint was restored satisfactorily and the shoulder joint function recovered normally; the chest CT showed no signs of recurrence and metastasis. However, there are still some pressing issues. For example, owing to the high cost of MR technology, it cannot be widely used at present. Additionally, wearing smart glasses causes discomfort and pain, which may not be conducive to the work of the surgeons. Lastly, current technology is not perfect for timely real‐time interaction, and image processing takes time, making delays are inevitable.

## 4 Conclusion

In this study, we used 3D reconstruction and MR technology to verify the feasibility of the reconstruction of the sternoclavicular joint with the proximal fibula before surgery, achieving satisfactory clinical results postoperatively. Although further research with a larger sample size is needed to confirm our findings, this case preliminarily suggests that 3D reconstruction and MR technology can help in the selection of surgical safe reconstruction methods for bone defects.

## Author Contributions

**Yuan Gao:** conceptualization, writing – original draft. **Feng Pan:** data curation, resources. **Jun Zhang:** formal analysis, resources. **Songjie Ji:** data curation, resources. **Guanning Shang:** writing – review and editing.

## Consent

Written informed consent was obtained from the patient and a family member for publication of this case report and accompanying images.

## Conflicts of Interest

The authors declare no conflicts of interest.

## Data Availability

The authors have nothing to report.

## References

[1] M. H. Priemel , N. Stiel , J. Zustin , A. M. Luebke , C. Schlickewei , and A. S. Spiro , “Bone Tumours of the Clavicle: Histopathological, Anatomical and Epidemiological Analysis of 113 Cases,” Journal of Bone Oncology 16 (2019): 100229. 30976505 10.1016/j.jbo.2019.100229 PMC6439286

[2] C. E. Nistor , A. Ciuche , A. P. Cucu , et al., “Clavicular Malignancies: A Borderline Surgical Management,” Medicina (Kaunas, Lithuania) 58, no. 7 (2022): 910. 35888630 10.3390/medicina58070910 PMC9315479

[3] Y. Liu , X.‐Y. Huang , W.‐Y. Feng , et al., “Analysis of the Clinical Efficacy of Tumor Resection Methods Used in 20 Patients With Clavicular Tumor,” World Journal of Surgical Oncology 17 (2019): 106. 31208415 10.1186/s12957-019-1642-4 PMC6580492

[4] R. L. Tate , M. Perdices , U. Rosenkoetter , et al., “The Single‐Case Reporting Guideline in BEhavioural Interventions (SCRIBE) 2016 Statement,” Journal of School Psychology 56 (2016): 133–142. 27268573 10.1016/j.jsp.2016.04.001

[5] B. Lin , Y. He , Y. Xu , and M. Sha , “Outcome of Bone Defect Reconstruction With Clavicle Bone Cement Prosthesis After Tumor Resection: A Case Series Study,” BMC Musculoskeletal Disorders 15 (2014): 183. 24885109 10.1186/1471-2474-15-183 PMC4046063

[6] R. M. Green , D. Waldman , K. Ouriel , P. Riggs , and J. A. DeWeese , “Claviculectomy for Subclavian Venous Repair Long‐Term Functional Results,” Journal of Vascular Surgery 32 (2000): 315–321. 10917992 10.1067/mva.2000.106949

[7] M. T. Provencher , D. L. Bernholt , L. A. Peebles , and P. J. Millett , “Sternoclavicular Joint Instability and Reconstruction,” Journal of the American Academy of Orthopaedic Surgeons 30, no. 16 (2022): e1076–e1083. 35502995 10.5435/JAAOS-D-19-00611

[8] P. Goetti , C. Pham , N. Gallusser , et al., “Total Clavicle Reconstruction With Free Peroneal Graft for the Surgical Management of Chronic Nonbacterial Osteomyelitis of the Clavicle: A Case Report,” BMC Musculoskeletal Disorders 20, no. 1 (2019): 211. 31084601 10.1186/s12891-019-2588-y PMC6515610

[9] N. G. Momberger , J. Smith , and D. A. Coleman , “Vascularized Fibular Grafts for Salvage Reconstruction of Clavicle Nonunion,” Journal of Shoulder and Elbow Surgery 9 (2000): 389–394. 11075322 10.1067/mse.2000.107090

[10] D. F. Kalbermatten , M. Haug , D. J. Schaefer , et al., “Computer Aided Designed Neo‐Clavicle out of Osteotomized Free Fibula: Case Report,” British Journal of Plastic Surgery 57 (2004): 668–672. 15380700 10.1016/j.bjps.2004.05.013

[11] J. Li , Z. Wang , J. Fu , L. Shi , G. Pei , and Z. Guo , “Surgical Treatment of Clavicular Malignancies,” Journal of Shoulder and Elbow Surgery 20 (2011): 295–300. 20797879 10.1016/j.jse.2010.05.009

[12] A. D. Deng , M. Innocenti , and R. Arora , “Vascularized Small‐Bone Transfers for Fracture Nonunion and Bony Defects,” Clinics in Plastic Surgery 47 (2020): 501–520. 32892797 10.1016/j.cps.2020.06.005

[13] L. Ye and G. I. Taylor , “A 10‐Year Follow‐Up of a Free Vascularized Fibula Flap Clavicle Reconstruction in an Adult Plastic and Reconstructive Surgery,” Global Open 5 (2017): e1317. 10.1097/GOX.0000000000001317 PMC5426893 28507874

[14] R. O'Neill , A. Skochdopole , A. E. Grush , et al., “Clavicular Reconstruction Utilizing Free Fibula Flap With Periosteal Extension,” Microsurgery 43 (2023): 157–160. 36541829 10.1002/micr.30995

[15] V. S. Devaraj , S. P. Kay , and A. G. Batchelor , “Vascularised Reconstruction of the Clavicle,” British Journal of Plastic Surgery 43 (1990): 625–627. 2224363 10.1016/0007-1226(90)90133-k

[16] J. Frost , J. Demke , W. Idicula , and M. Diab , “Surgical Correction of Torticollis due to Agenesis of the Sternocleidomastoid: Case Study and Review,” Cleft Palate‐Craniofacial Journal 57 (2020): 520–523. 10.1177/1055665619887315 31726869

[17] J. C. Katthagen , D. C. Marchetti , K. D. Dahl , T. L. Turnbull , and P. J. Millett , “Biomechanical Comparison of Surgical Techniques for Resection Arthroplasty of the Sternoclavicular Joint,” American Journal of Sports Medicine 44 (2016): 1832–1836. 27159312 10.1177/0363546516639302

[18] R. Moreta‐Martinez , A. Pose‐Díez‐de‐la‐Lastra , J. A. Calvo‐Haro , L. Mediavilla‐Santos , R. Pérez‐Mañanes , and J. Pascau , “Combining Augmented Reality and 3D Printing to Improve Surgical Workflows in Orthopedic Oncology: Smartphone Application and Clinical Valuation,” Sensors 21 (2021): 1370. 33672053 10.3390/s21041370 PMC7919470

[19] P. Stefan , M. Pfandler , P. Wucherer , et al., “Team Training and Assessment in Mixed Reality‐Based Simulated Operating Room: Current State of Research in the Field of Simulation in Spine Surgery Exemplified by the ATMEOS Project,” Der Unfallchirurg 121 (2018): 271–277. 29546445 10.1007/s00113-018-0467-x

[20] V. Chakravarthy , S. Sheikh , E. Schmidt , and M. Steinmetz , “Imaging Technologies in Spine Surgery,” Neurosurgery Clinics of North America 31, no. 1 (2020): 93–101. 31739935 10.1016/j.nec.2019.08.011

[21] X. Wu , R. Liu , J. Yu , et al., “Mixed Reality Technology‐Assisted Orthopedics Surgery Navigation,” Surgical Innovation 25 (2018): 304–305. 29701134 10.1177/1553350618771413

[22] P. F. Lei , S. L. Su , L. Y. Kong , C. G. Wang , D. Zhong , and Y. H. Hu , “Mixed Reality Combined With Three‐Dimensional Printing Technology in Total Hip Arthroplasty: An Updated Review With a Preliminary Case Presentation,” Orthopaedic Surgery 11 (2019): 914–920. 31663276 10.1111/os.12537 PMC6819179

